# Kinetic and computational molecular docking simulation study of novel kojic acid derivatives as anti-tyrosinase and antioxidant agents

**DOI:** 10.1080/14756366.2019.1609467

**Published:** 2019-05-10

**Authors:** Yan-Mei Chen, Chen Li, Wen-Jing Zhang, Yan Shi, Zi-Jie Wen, Qing-Xi Chen, Qin Wang

**Affiliations:** School of Life Sciences, Xiamen University, Xiamen, China

**Keywords:** Kojic acid derivatives, tyrosinase inhibition, antioxidant

## Abstract

The novel kojic acid derivatives KAD1 and KAD2 have been demonstrated that they exhibited potent anti-melanogenesis activity in our previous report. In this study, we further study the inhibitory mechanism on mushroom tyrosinase. The inhibitory types of both KADs on diphenolase were classified as mixed type based on the results of the kinetic model. The interaction between KADs and tyrosinase was illustrated by fluorescence quenching, molecular docking and copper chelate activity. The KADs were also evaluated with respect to their antioxidant activities by DPPH and ABTS^+^ assays. The results showed that KADs have more potent antioxidant activities than kojic acid. Our study could provide new ideas for the development of new anti-tyrosinase and antioxidant agents.

## Introduction

Tyrosinase (EC 1.14.18.1) is a copper-containing enzyme widely occurred in bacteria, fungi, plants and animals.^1^ Tyrosinase plays a crucial role in the biosynthesis process of melanin pigment.^2^ The biosynthetic route mainly carries out two following different metabolisms for melanin formation. First, tyrosinase catalyses the hydroxylation of l-tyrosine which is defined as monophenolase activity, and the second, the enzymatic oxidation of l-DOPA to o-quinone is defined as diphenolase activity. In the process of melanogenesis, dopaquinone rapidly and spontaneously evolves towards the formation of various melanin pigments. Therefore, tyrosinase plays a crucial role in regulating the metabolic pathway of melanin formation. In the human skin cells, tyrosinase is the only rate-limiting enzyme for melanogenesis and the expression of tyrosinase has physiological functions in the process of melanoma’s occurrence and development. Abnormal expression of tyrosinase can directly or indirectly lead to skin diseases, such as vitiligo, malignant melanoma and freckl.^3^^,^^4^ To prevent and treat these diseases, many researchers have made great efforts to exploit and develop the tyrosinase inhibitors such as kojic acid, arbutin and so on. Tyrosinase inhibitors are usually obtained from natural product and synthetic chemical compounds. In comparison to the chemically synthesized compounds, the natural products have gained more attention due to their increasing demands in cosmetics. The advantages of sustainable and nontoxic are taken into account in developing UV-protective, anti-aging and skin-whitening products for skin and hair. Some of the natural products such as alkaloids, flavonoids, tannins, saponins, *Polyalthia longifolia* and coumarins are studied.^5^ On the other hand, people have gradually shifted their attention to chemical tyrosinase inhibitors. With the help of current accessible methods to rationally design efficient synthetic derivatives, many synthetic tyrosinase inhibitors have been prepared by modifying the various structural moieties of natural tyrosinase inhibitors. Many researches showed that tyrosinase inhibitors also possess antioxidant, antibacterial and antifungal properties which play a crucial role in the treatment of skin diseases.^6^ The process of oxidative degradation, particularly that induced by reactive oxygen species (ROS), may lead to oxidative stress, which is an imbalance between antioxidant systems and the production of oxidants.^7^ Oxidative stress is associated with many diseases, such as aging and cancer.^8^^,^^9^ Antioxidants play a significant role in reducing aging effects, and thus the antioxidant property in designing whitening agent is highly considered.

In our previous study,^10^ the novel kojic acid derivatives KAD1 and KAD2 were synthesized and we found that they strongly inhibited the diphenolase activity of mushroom tyrosinase with IC_50_ of 8.33 and 7.50 μM, respectively, whose inhibitory effects were much better than kojic acid. Particularly, we found KAD2 could effectively inhibit melanogenesis on B16F10 cell by suppressing the expression of the phosphorylation of protein kinase A (PKA) and cAMP-response element binding protein (CREB) and activating the phosphorylation of Akt. In this work, we further studied their inhibitory kinetics and molecular simulation on mushroom tyrosinase. Experiments concerning kinetic analysis, fluorescence quenching technique, copper chelate activity and molecular docking were carried out to illustrate the inhibition mechanism on tyrosinase. Additionally, antioxidant capacities also were determined.

## Materials and methods

### Chemicals

Mushroom tyrosinase (EC 1.14.18.1), 3,4-dihydroxyphenylalanine (l-DOPA), l-tyrosine (l-Tyr), dimethylsulfoxide (DMSO), 2,2-diphenyl-1-picrylhydrazyl (DPPH) and kojic acid were all purchased from Sigma-Aldrich (St. Louis, MO, USA). All commercially available starting materials and reagents were used without further purification. Other reagents were of analytical grade. The water used was redistilled and ion free.

### Enzyme assay

The assay was conducted following the method of Cui et al.^11^ In this reaction, l-Tyr and l-DOPA were used as the substrate for monophenolase and diphenolase activity assay, respectively. The reaction system (3 ml) contained 1 mM l-Tyr or 0.5 mM l-DOPA and enzyme in 50 mM Na_2_HPO_4_–NaH_2_PO_4_ buffer (pH 6.8) and different concentrations of KADs. The KADs were dissolved in DMSO and diluted to appropriate concentration, such that the final concentration of DMSO is maintained at 3.3%. The controls without inhibitor but containing 3.3% DMSO in the reaction media were routinely carried out, while kojic acid was as positive control. The enzyme activity was evaluated by increasing the absorbance at 475 nm accompanying the oxidation of the substrates with a molar absorption coefficient of 3700 (M^−1 ^cm^−1^). The inhibition types of KADs on the enzyme were determined using Lineweaver–Burk plots and the inhibition constants of kinetic equations were obtained from secondary plots of the apparent *K*_m_/*V*_max_ or 1/*V*_max_ versus the concentrations of the inhibitor. The reaction was carried out at 37 °C. A Thermo MULTISKAN GO spectrophotometer was used for absorbance and kinetic measure.

### Fluorescence quenching

Fluorescence spectra were determined as previously reported by Zhu.^12^ Cary Eclipse fluorescence spectrophotometer was used to record the fluorescence intensities at an excitation wavelength of 280 nm, and emission and excitation slit widths were 10 nm and 5 nm, respectively. At wavelengths of 300–450 nm, fluorescence emission spectra were recorded. In this assay, 10 μL of KADs of different concentrations were incorporated with 2 ml tyrosinase solution (0.2 mg/ml).

The fluorescence quenching data expressed as the fluorescence intensity against KADs concentration. Fluorescence quenching was calculated using the Stern–Volmer equation:

$$F_{0}/F\;=\;\text{1}\;+\;K_{\text{SV}}\left[Q\right]$$

Where *F*_0_ is the fluorescence intensities of tyrosinase, *F* is the fluorescence intensities after addition of the quencher, [*Q*] is the concentration of the quencher and *K*_SV_ is the Stern–Volmer quenching constant. For the calculation of biomolecular quenching constants, the data were plotted as *F*_0_/*F* against [*Q*], with the biomolecular quenching constants calculated by linear regression.

There are two known fluorescence quenching mechanisms: static quenching is caused by complex formation while dynamic quenching is caused by collisional processes.^13^ For the static quenching, the *K*_SV_ value must be larger than 100 M^−1^ and the apparent binding constant (*K*_A_) and the binding sites (*n*) were estimated by plots of log[(*F*_0_−*F*)/*F*] versus log[*Q*] using the following equation:^14^

$$\text{log }\left(\left(F_{0}\;-\;F\right)/F\right)\;=\;\text{log}K_{\text{A}}\;+\;n\text{log }\left[Q\right]$$

### *In silico* docking of tyrosinase

Docking simulation was further conducted supporting anti-tyrosinase activities of KADs to clarify the molecular mechanism by Molecular Operation Environment 2008 software (MOE). The structure of tyrosinase from *Agaricus bisporus* PPO3 (PDB: 2Y9W) was downloaded from PDB database.^15^ During the computer docking, the caddie protein, the water molecules and the exogenous ions of the enzyme structure are removed first. The 3 D structure of KADs were built by ChemDraw 14.0 and saved as a mol format. Both the tyrosinase and the inhibitor were exported to MOE. The structures of tyrosinase and KADs were energy minimized with MM^+^ force field until the root mean square gradient of 0.05 Kcal/Ǻ mol is obtained before docking. And then, choose the site which is closest to active centre. In the process of molecular docking, the receptor and site were set to receptor atoms and dummy atoms, respectively. The refinement was set to force field, the retain value was set to 20 and the rescoring 1 and 2 were set to London dG.^16^

To investigate whether the binding of copper ions with the inhibitor takes place at the active site of tyrosinase, the assay was carried out according to the method by Ismaya with some modifications.^17^ Stock solutions of each KADs (10 mM) were prepared in DMSO. Then, 0.33 mM solutions were prepared in a cuvette containing phosphate buffer (10 mM, pH 7.4), and treated with different concentrations of CuCl_2_ (0, 60, 120, 180 or 240 µM). After 10 min of incubation period at room temperature, absorbance was scanned from 240 to 600 nm. Moreover, the relative activity of tyrosinase was determined by adding 0.1 ml various concentrations of CuCl_2_–KADs complexes from the previous step. The reaction system and the method were the same as the determination of diphenolase activity.

### Antioxidant activity analysis

The antioxidant capacities of compounds usually analysed through DPPH and ABTS^+^ free-radical scavenging assays. The DPPH free-radical scavenging was conducted according to the procedure described by Chen.^18^ Stock solution (0.2 mM DPPH) was prepared in 95% methyl alcohol and diluted to with OD value of 1.1 ± 0.05 at 517 nm for analysis. Briefly, 0.1 ml of compounds dissolved in DMSO at different concentrations (50, 200, 400 and 800 μg/ml) were added to DPPH with a methyl alcohol solution (0.2 mM, 2.9 ml). The mixture was shaken gently and absorbance was read at 517 nm every 5 min, a total of 30 min. The ABTS^+^ free-radical scavenging activity assay was performed as described by Ai^9^ with slight modification. 7 mM ABTS^+^ and 2.45 mM K_2_S_2_O_8_ were dissolved in 10 mM sodium phosphate buffer and then placed at dark room for 16 h to obtain ABTS^+^ stock solution. ABTS^+^ solution for analysis was prepared by diluting its stock solution with 80% ethanol to with OD value of 0.7 ± 0.05 at 734 nm. The tested compound (0.1 ml) was added to the ABTS^+^ free-radical solution (2.9 ml) and mixed thoroughly. After 6 min of incubation period at room temperature, absorbance was read at 714 nm. The scavenging capacity of the antioxidant for the free-radical was calculated according to the following equation:

$$\text{Radical scavenging effect }\left(\%\right)\;=\;\left(\text{1}\;-\;A_{\text{1}}/A_{0}\right)\;\times \;\text{1}00\%$$

where *A*_0_ is the absorbance of blank control and *A*_1_ is the absorbance in the presence of sample. Data are represented from three independent experiments, and the mean values were calculated. Ascorbic acid (V_c_) was used as a positive control.

### Statistical analysis

Data are presented as mean ± SD for triplicate determinations. Analysis of variance (ANOVA) followed by Duncan^a^ test were conducted to identify significant differences between samples using SPSS statistics 19 software. A value *p* < .05 was considered a significant difference.

## Results

### Inhibition effects of KADs on monophenolase activity of mushroom tyrosinase

**Figure 1.**
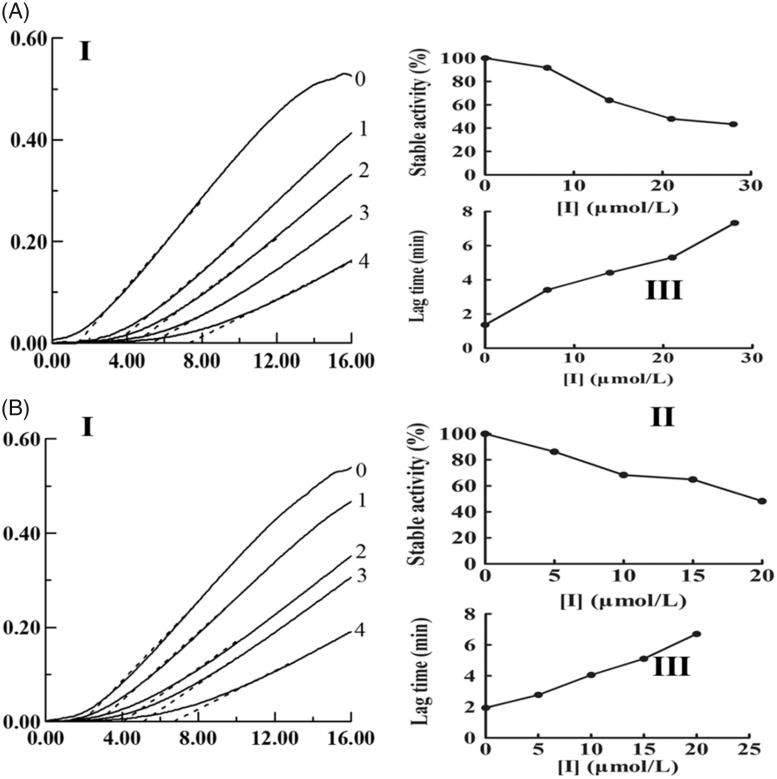
Effects of KADs on the monophenolase activities of tyrosinase. A and B represent KAD1 and KAD2, respectively. (I) Progress curves for the oxidation of l-Tyr by the enzyme. (II) Effects on the stable activity of l-Tyr by the enzyme. (III) Effects on the lag time of monophenolase activities of tyrosinase. The final concentrations of KAD1 for curves 0–4 were 0, 7, 14, 21 and 28 μM. The final concentrations of KAD2 for curves 0–4 were 0, 5, 10, 15 and 20 μM.

With different concentrations of KADs and using l-Tyr as the substrate, the monophenolase activity of mushroom tyrosinase was assayed. KAD1 and KAD2 all exhibited potent inhibition on tyrosinase activity. As presented in Figure 1, the steady-state rate of monophenolase activity was decreased and the lag time was increased with increasing of the concentrations of KADs. The compound KAD2 exhibited stronger inhibitory effect than KAD1 on monophenolase. The lag time was up to 6.7 min and the steady-state activity decreased to 48.29% by KAD2 with the maximum concentration (20 μM).

### Inhibition effects of KADs on diphenolase activity of mushroom tyrosinase

**Figure 2.**
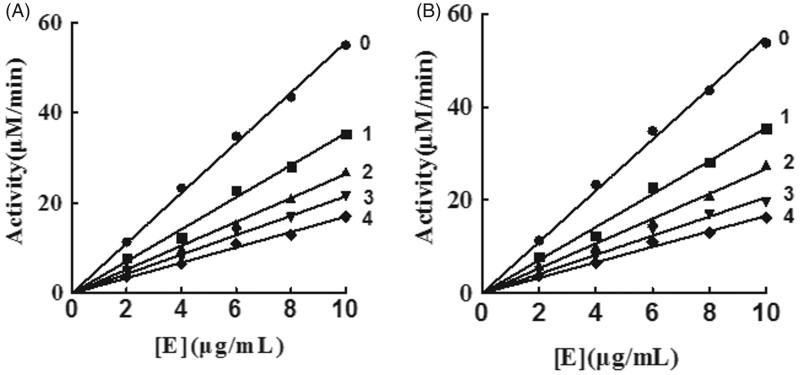
Inhibitory mechanisms of KADs on mushroom tyrosinase. A and B represent KAD1 and KAD2, respectively. The concentrations of KAD1 for curves 0–4 were 0, 5.83, 11.67, 17.50 and 23.33 μM, respectively. The concentrations of KAD2 for curves 0–4 were 0, 3.75, 7.50, 11.25 and 15.00 μM, respectively.

**Figure 3.**
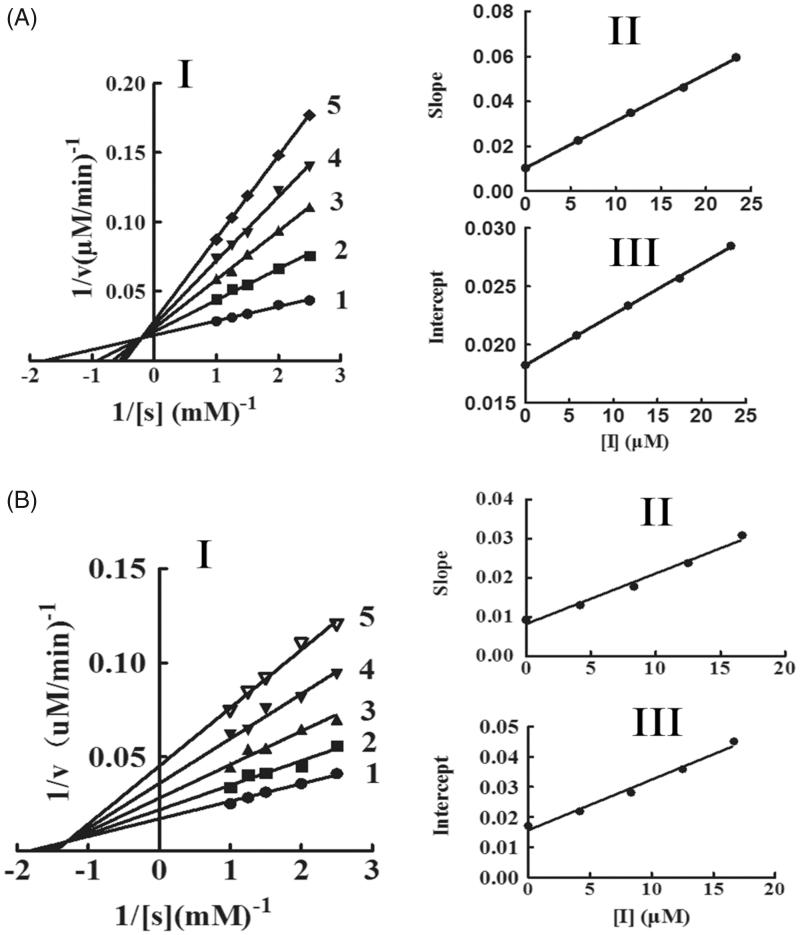
The inhibitory types and constants of KADs on tyrosinase. A and B represent KAD1 and KAD2, respectively. The concentrations of KAD1 for curves 1–5 were 0, 5.83, 11.67, 17.50 and 23.33 0 μM. The concentrations of KAD2 for curves 1–5 were 0, 3.75, 7.50, 11.25 and 15.00 μM, respectively.

The oxidation of l-DOPA by tyrosinase was studied to ascertain the inhibitory behaviour of KADs. The relationship between the remaining enzyme activity and enzyme concentration in the presence of different concentrations of KADs was a family of straight lines that all pass through the origin (Figure 2). Increasing the inhibitor concentration resulted in lowering of the slope of the line, indicating that it is the reversible property of the inhibition by KADs. The outcomes showed that they could not deactivate permanently the enzyme to decrease the amount of active enzyme in the presence of KADs, merely depressed the enzyme activities. Lineweaver–Burk plot analysis (the plots of 1/*v* vs. 1/[l-DOPA]) gave a family of straight lines with different slopes that intercept in the second quadrant, indicating that two compounds can bind not only with free enzyme but also with the enzyme–substrate complex (Figure 3). The results demonstrated that KADs were classified as mixed type inhibitors. The inhibition constant (*K*_I_) was obtained from the plot of the slopes versus the concentrations of KADs. The enzyme–substrate complex (*K*_IS_) was also obtained from the plot of the vertical intercepts versus the concentrations of KADs, as shown in Table 1. In the case, the values of *K*_IS_ are larger than *K*_I_.

**Table 1.** Inhibition constants of KADs on tyrosinase.

Sample	IC_50_ (μM)^10^		Inhibition		Inhibition constants (μM)	
	Monophenolase	Diphenolase	Mechanism	Type	*K*_I_	*K*_IS_
KAD1	21.58	8.33 ± 0.22	Reversible	Mixed	4.95	45.50
KAD2	20.51	7.50 ± 0.13	Reversible	Mixed	6.30	9.18
Kojic acid	ND	19.50 ± 0.68	Reversible	Competitive^11^	ND	ND

ND: not determined.

### Intrinsic fluorescence quenching analysis

**Figure 4.**
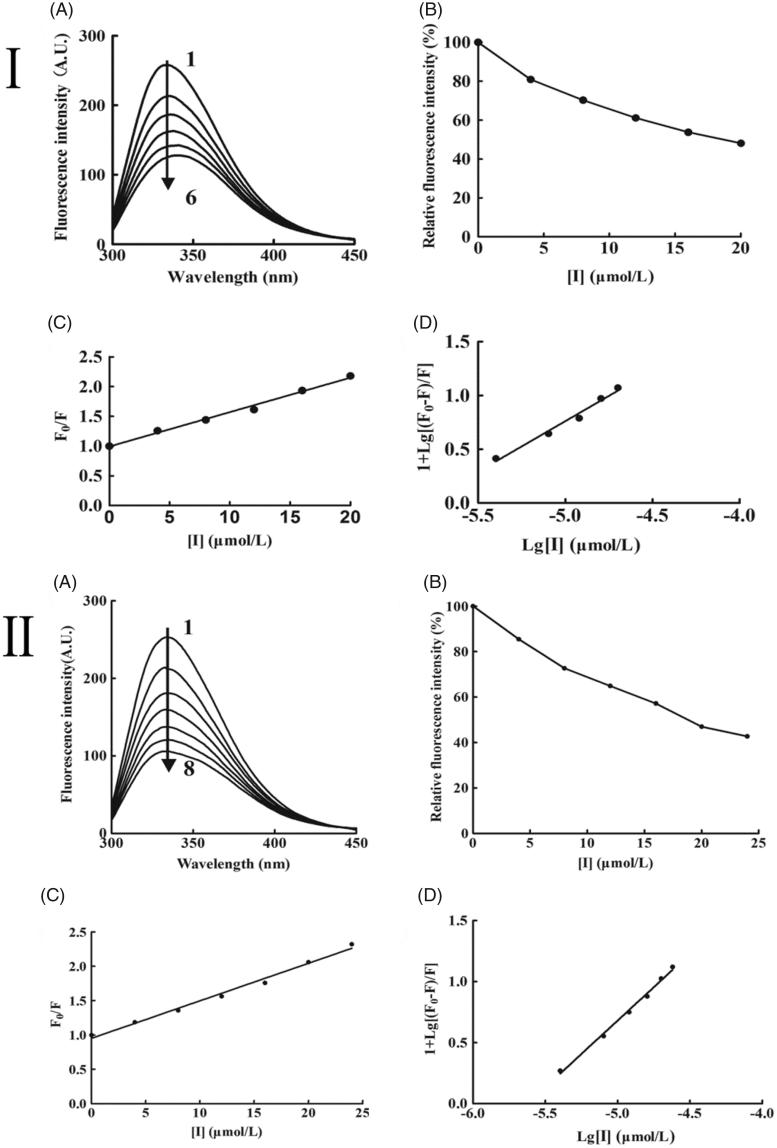
Variations in the intrinsic tyrosinase fluorescence with increasing the concentrations of KADs at *λ*_ex_ =280 nm. (A) Emission spectra of tyrosinase. (B) The quenching effect of tyrosinase. (C) The plot of [*F*_0_/*F*] against [I]. (D) The plot of lg[(*F*_0_−*F*)/*F*] against lg[*I*]. I and II represent KAD1 and KAD2, respectively. The concentrations of KAD1 for curves 1–6 were 0, 4, 8, 12, 16 and 20 μM. The concentrations of KAD2 for curves 1–7 were 0, 4, 8, 12, 16, 20 and 24 μM.

Intrinsic fluorescence quenching of protein was applied to retrieve many drug–protein binding information.^18^ The tryptophan residues of tyrosinase can be analysed under 280 nm of *λ*_ex_, and has a strong fluorescence in 330 nm of *λ*_em_. Tyrosinase exhibited a strong emission peak at 330 nm. With the increasing concentration of KADs, the fluorescence intensity of tyrosinase at 330 nm was gradually quenched, and the emission peak was changed from 330 to 340 nm with a slightly red shift, as seen in Figure 4(A,B). The results suggested that KADs might have interacted with tyrosinase and caused a conformational change of tyrosinase.

The mechanism of fluorescence quenching was usually classified as static quenching and dynamic quenching.^19^ The fluorescence data were determined using the Stern–Volmer equation to distinguish these two quenching mechanisms. The plot of lg[(*F*_0_−*F*)/*F*] against lg[KADs] for tyrosinase with various concentrations of KADs (Figure 4(C,D)). They presented a good linear relationship, indicating that the fluorescence quenching procedure was single type. As shown in the Table 2, *K*_SV_, *K*_A_ and *n* were obtained, all the results suggested that the fluorescence quenching of tyrosinase by KADs were a static quenching process.

**Table 2.** Stern–Volmer equation for the interaction between tyrosinase and KADs.

Inhibitor	Type of quenching	*K*_SV_ (M^−1^)	*K*_A_ (M^−1^)	*n*
KAD1	Static	5.84 × 10^4^	2.87 × 10^4^	0.9394
KAD2	Static	5.70 × 10^4^	1.41 × 10^5^	1.0394

### Molecular docking and copper chelate activity

**Figure 5.**
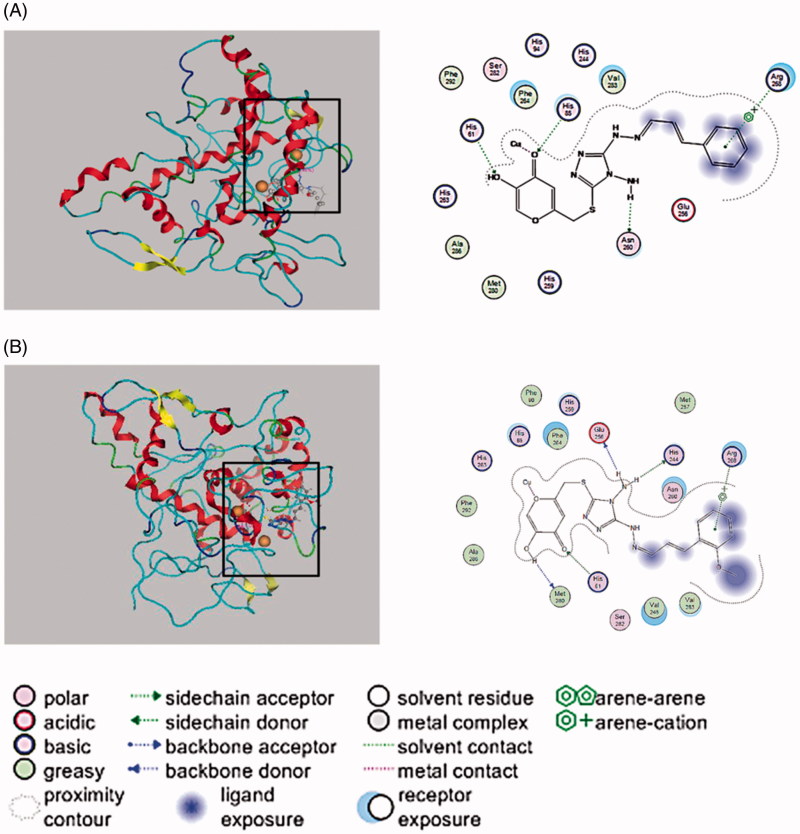
Interaction between KADs and the tyrosinase analysed by molecular docking. In the graph, the size and intensity of the turquoise discs, surrounding the tyrosinase residues, presented different exposures which were changed by kojic acid derivatives. A and B represent KAD1 and KAD2, respectively.

Docking simulation was further performed to clarify the inhibitory mechanism of KADs on tyrosinase. Chelation of tyrosianse with two copper ions (Cu^2+^) by His61, His85, His94, His259, His263 and His294, spatially oriented within the activate dioxygen in the active site to facilitate dioxygen to initiate catalytic activity.^20^ As shown in Figure 5, the docked conformation revealed that three compounds all docked inside the binding regions of tyrosinase. Due to the structural similarity of the compounds, they exhibited a similar interaction with tyrosinase. The phenyl moiety of the two compounds were able to interact with Arg268. For KAD1, the amino group was able to interact with the side chain acceptor of Asn260, and the hydroxyl group interacted with the side chain donor of His61. Especially, the carbonyl could interact with the copper and the side chain donor of His85 which coordinately bound to the copper ions. It can be seen clearly that KAD2 also could directly interact with a copper, Met280 could act on the hydrogen atom of hydroxyl, the oxygen atoms of ketone was able to interact with the side chain acceptor of His61 and amino hydrogen could interact with Glu256 and His244.

**Figure 6.**
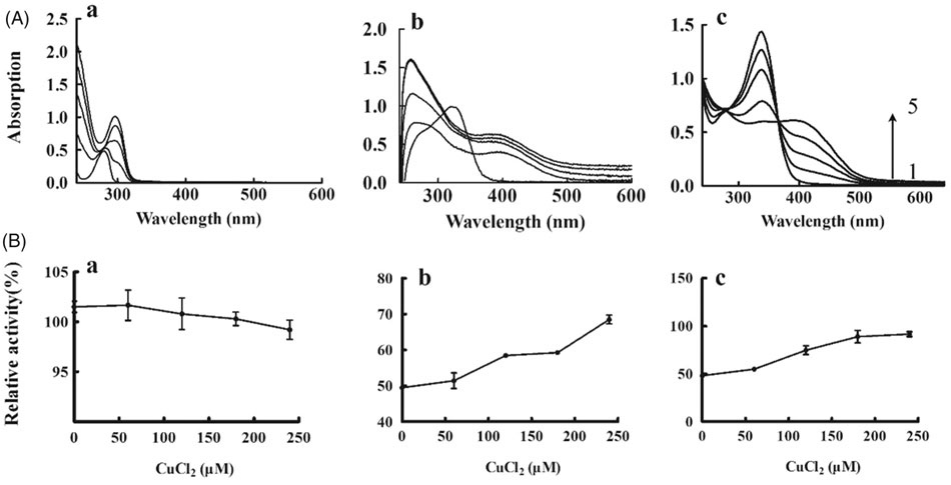
Copper chelate activity of KADs. (A) Absorption spectra for the Cu–KADs complex; (B) Tyrosinase activity. a, b and c represent L-DOPA, KAD1 and KAD2, respectively. The concentrations of CuCl_2_ for curves 1–5 were 0, 60, 120, 180 and 240 μM, respectively. *n* = 3, error bars, mean ± SD.

The structural correspondence between KADs and copper–KAD complex is supported through collecting the spectra treated with increasing concentrations of copper ion. The previous study have reported that there was a red shift of peak for l-DOPA presenting the possibility of copper chelation during interaction with Cu^2+^ ions.^21^ As shown in Figure 6(A)-a, the l-DOPA has a red shift of peak (from 260 to 290 nm) with increasing concentrations of Cu^2+^, which was consistent with the published results. The same analysis for KAD1 was shown in Figure 6(A)-b. The results showed that the peak of 340 nm disappeared and a new peak of 390 nm increased during interaction with the Cu^2+^, which indicated that KAD1 might interact with Cu^2+^ forming new complexes. Similarly, KAD2 had an absorption peak at 340 nm. With an increase of the concentration of Cu^2+^, the peak intensity of 340 nm decreased or even disappeared and the new peak intensity of 390 nm increased, as shown in Figure 6(A)-c. On interaction with Cu^2+^, KAD1 and KAD2 showed significant red shift peaks which were assumed to be a characteristic of copper chelation. To further verify the results, the effects of copper–KADs chelation on diphenolase activity were measured. The results were shown in Figure 6(B). For the DOPA (Figure 6(B)-a), there is no obvious change of the relative tyrosinase activities while improved for KAD1 and KAD2 with increasing concentrations of Cu^2+^.

### The antioxidant activities of KADs

**Figure 7.**
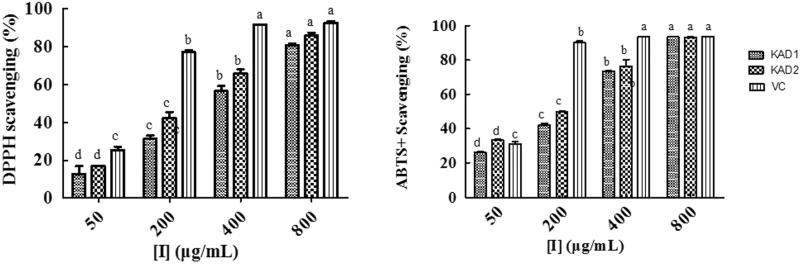
DPPH and ABTS^+^ free-radical scavenging capacity of KADs. The data are presented as percentages compared with the control group (set to 100%) and represented as the mean ± SD, *n* = 3. Different alphabetical letters indicate significant differences among the conditions as assessed by one-way ANOVA followed by the Duncan^a^ test (*p* < .05).

In this study, DPPH and ABTS^+^ free-radicals scavenging assays were done to determine the antioxidant capacities of KADs. As shown in Figure 7, KADs showed more potent antioxidant activity than kojic acid, but weaker than V_c_. KAD2 showed the better ability to reduce the free radical with IC_50_ of DPPH was 227.80 μg/ml and IC_50_ of ABTS^+^ was 126.63 μg/ml.

## Discussion

Tyrosinase has been proven to have an important effect on different species: for the microorganism, tyrosinase is related to the stress resistance and has been predicted as potential target of antiseptics; for the insects, tyrosinase participates the growth process and play important roles in tanning and exoskeleton process.^22^ In this article, the inhibitory mechanism on mushroom tyrosinase have been studied and evaluated by novel kojic acid derivatives bearing Schiff base. In general, inhibitors of tyrosinase can be classified into four categories: competitive, non-competitive, uncompetitive and mixed type.^23^ Due to the two new compounds have similar structure, there are slight differences on inhibition effect between them and the same inhibition mechanism was showed. KADs were found to inhibit both the monophenolase and diphenolase activity of mushroom tyrosinase. For monophenolase activity, KADs were able to inhibit the steady-state activity and extend the lag time. For diphenolase activity, they belonged to mixed type inhibitor which bind to both free enzyme and enzyme–substrate complex. The inhibitory mechanism of KADs were reversible demonstrated that the inhibitor could not lessen the amount of the efficient enzyme, but just decreased the activity of tyrosinase. The inhibition kinetics analysed by Lineweaver–Burk plots showed that the compounds acted as a mixed inhibitor of mushroom tyrosinase. Moreover, the *K*_IS_ values larger than *K*_I_ indicating the affinity of enzyme–substrate complex for free enzyme is weaker than that of the inhibitors. Fluorescence quenching was used to investigate the interactions of ligands and proteins.^24^ The presented results indicated that the tyrosinase fluorescence intensity decreased in the presence of KADs. It suggested that the structure of tyrosinase was changed on addition of KADs. The values of Stern–Volmer quenching constant (*K*_SV_) for the two compounds were all larger than 100 M^−1^ and indicated there was the static mechanism of fluorescence quenching between tyrosinase and KADs.^25^

The results of molecular docking by MOE suggested that KAD1 and KAD2 not only formed hydrogen bonds with tyrosinase but also could bind directly to a copper ion at the active site through compounds with molecular docking simulation. What is more, KAD2 was more closely and tightly closed to the active site of the enzyme, thus it had more potent inhibitory activity against tyrosinase. These results were consistent with IC_50_ values. The results of copper chelate activity showed that a copper–KAD chelate was formed for KAD1 and KAD2. It also demonstrated that KAD1 and KAD2 could form metal interaction with tyrosinase. Furthermore, the relative tyrosinase activities in the presence of KADs were studied after treated with different concentrations of Cu^2+^. The results showed that the relative tyrosinase activities were almost no changes for DOPA while increased by 1.5 times for KAD1 and 2 times for KAD2. It is suggested that the copper chelate could not affect the tyrosinase activity and the increase of tyrosinase activity for KAD1 and KAD2 was due to the decrease of available inhibitor concentration, which was caused by the formation of chelate, these results were consistent with Cui et al.^12^ Kojic acid and some kojic acid derivatives have been found to inhibit tyrosinase by chelating the copper ion which normally present in active site of tyrosinase.^26^ In conclusion, the kojic acid derivatives could inhibit the activity of tyrosinase in two ways. First, the inhibitor combined with enzyme to affect the microenvironment which was very close to the active site of tyrosinase. The second way was enzyme, inhibitor and the substrate to form ternary compound, and inhibitor could prevent the oxidative process of substrate, which brings down the formation of melanin.

Most of the compounds with antioxidant properties are from natural product. Many kinds of plants have been reported that have good antioxidant properties such as flavonoid, olive, tea and so on.^27^ Kojic acid also was known as a scavenger of free radicals.^28^ The antioxidant activity of two compounds were determined by DPPH and ABTS^+^ free-radical scavenging assay. The results showed that KADs have good antioxidant activity. Reactive compounds may be oxygenated/nitrogenated free radicals defined as chemical compounds possessing an unpaired electron.^27^ Phenolic compounds contain hydroxyl groups, which can donate H atom which made them possess good antioxidant activities.^8^ Radicals are most commonly quenched by two mechanisms, transfer of either a hydrogen atom or an electron to convert the radical to a stable species: hydrogen atom transfer and single electron transfer.^29^ The antioxidant mechanism of KADs are based on its chemical structures. KADs contain amidogen and hydroxyl groups, which can transfer electron to DPPH or ABTS^+^. Interestingly, many compounds have been reported they possess anti-tyrosinase properties while also possess good antioxidant activities.^30^ It seems that there are some relationships between them which need further study.

In conclusion, KADs significantly inhibited tyrosinase and well reduced the free radical. Our study would lay scientific foundation for their utilization in designing of new anti-tyrosinase and anti-oxidant agents.
